# Quercetin delays postovulatory aging of mouse oocytes by regulating SIRT expression and MPF activity

**DOI:** 10.18632/oncotarget.16219

**Published:** 2017-03-15

**Authors:** HaiYang Wang, Yu-Jin Jo, Jeong Su Oh, Nam-Hyung Kim

**Affiliations:** ^1^ Department of Animal Sciences, Chungbuk National University, Cheongju, Korea; ^2^ Department of Genetic Engineering, College of Biotechnology and Bioengineering, Sungkyunkwan University, Suwon, Korea

**Keywords:** MPF, oocyte, postovulatory aging, quercetin, SIRT

## Abstract

If no fertilization occurs at an appropriate time after ovulation, oocyte quality deteriorates rapidly as a process called postovulatory aging. Because the postovulatory aging of oocytes has detrimental effects on embryo development and offspring, many efforts have been made to prevent oocyte aging. Here we showed that quercetin prevented the decline in oocyte quality during postovulatory aging of oocytes. Quercetin treatment reduced aging-induced morphological changes and reactive oxygen species accumulation. Moreover, quercetin attenuated the aging-associated abnormalities in spindle organization and mitochondrial distribution, preventing decrease of SIRT expression and histone methylation. Quercetin also ameliorated the decrease in maturation-promoting factor activity and the onset of apoptosis during postovulatory aging. Furthermore, quercetin treatment during postovulatory aging improves early embryo development. Our results demonstrate that quercetin relieves deterioration in oocyte quality and improves subsequent embryo development.

## INTRODUCTION

Mammalian oocytes are arrested at the metaphase of the second meiosis (MII) stage and are awaiting fertilization. If no fertilization occurs for a prolonged period, oocytes progressively undergo a time-dependent deterioration in quality, referred to as “postovulatory aging” [1]. Numerous studies have shown that postovulatory aging is associated with a range of defects, including abnormalities in structures of the zona pellucida, oolemma, cortical granules, mitochondria, and meiotic spindle and chromosome organizations [2–7]. Postovulatory aging is also accompanied by diverse biochemical and molecular changes, such as generation of reactive oxygen species (ROS), reduced maturation-promoting factor (MPF) activities, a decrease in the expression of anti-apoptotic factor BCL-2, activation of caspase-3, and changes in epigenetic modifications [8–11]. These aging-induced morphological, cellular, and molecular changes have the potential to impair fertilization and subsequent embryo development.

Quercetin is a plant-derived flavonoid present in many edible fruits and vegetables. Numerous reports have shown that quercetin exerts antioxidant, anti-apoptotic, and anti-inflammatory effects and protects various types of cells or tissues against oxidative stress [12–15]. Moreover, recent studies have shown that quercetin improves porcine oocyte nuclear maturation and subsequent embryo development by reducing ROS levels [16, 17]. More recently, quercetin has been shown to protect mouse embryos from oxidative injury [18].

Although the antioxidant effects of quercetin have been well characterized in certain types of somatic cells, including early embryos, no studies have yet investigated the effect of quercetin on postovulatory oocyte aging. In the present study, we investigated whether quercetin can protect oocytes from postovulatory aging, and explored the underlying cellular and molecular mechanisms of these effects.

## RESULTS

### Quercetin reduces morphological changes associated with postovulatory oocyte aging

**Figure 1:**
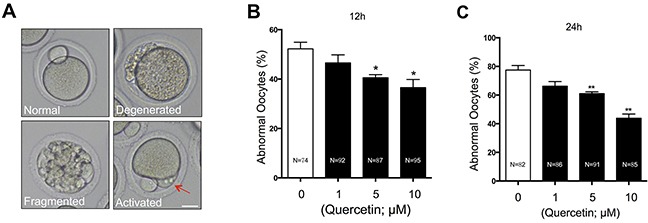
Effects of quercetin on oocyte morphology during postovulatory aging **(A)** Representative morphologies of oocytes after *in vitro* culture are shown. Postovulatory aged oocytes showed various morphological defects, including degeneration, fragmentation, and abortive spontaneous egg activation evidenced by initiation of extrusion of second polar body (red arrow). Scale bar: 20 μm. Oocytes with abnormal morphologies were quantified after culturing for 12 **(B)** or 24 **(C)** h with 0, 1, 5, and 10 μM quercetin. *p < 0.05, **p < 0.01.

To explore the potential involvement of quercetin in aging-induced morphological changes, we treated MII oocytes cultured *in vitro* with 0 (control), 1, 5, or 10 μM quercetin. After 12 h and 24 h of aging, the control oocytes showed various time-dependent morphological defects, including degeneration, fragmentation, and parthenogenetic activation (Figure 1A). When MII oocytes were exposed to 1 μM quercetin, the rate of morphological defects was not significantly lower compared with control oocytes (Figure 1B and 1C). However, the rate of morphological defects was significantly decreased after treatment with 5 or 10 μM quercetin. The preventive effects of quercetin on aging-induced morphological changes were dose-dependent. These findings suggest that quercetin prevents postovulatory aging in mouse oocytes.

### Quercetin decreases ROS accumulation in aging oocytes

**Figure 2:**
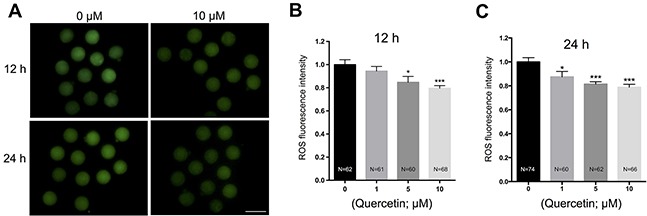
Effects of quercetin on ROS generation in aging oocytes **(A)** Representative images of H2DCFDA fluorescence in 0 and 10 μM quercetin-treated oocytes. Scale bar: 100 μm. After culturing MII oocytes for 12 h **(B)** or 24 h **(C)** in the presence of 0, 1, 5, and 10 μM quercetin, quantitative analysis of fluorescence intensity was conducted. *p < 0.05, ***p < 0.001.

Oxidative stress has been considered as a key mechanism underlying cellular aging [7]. Therefore, we examined whether quercetin protects oocytes from oxidative stress during postovulatory aging. We measured intracellular ROS levels after 12 h and 24 h of culture. Following quercetin treatment, oocytes showed a significant dose-dependent decrease in ROS accumulation (Figure 2A–2C). Because 10 μM quercetin showed the best preventive effects on aging-induced morphological changes and ROS accumulation, we used this concentration in all subsequent experiments.

### Quercetin prevents oocyte aging by delaying downregulation of SIRT expression

**Figure 3:**
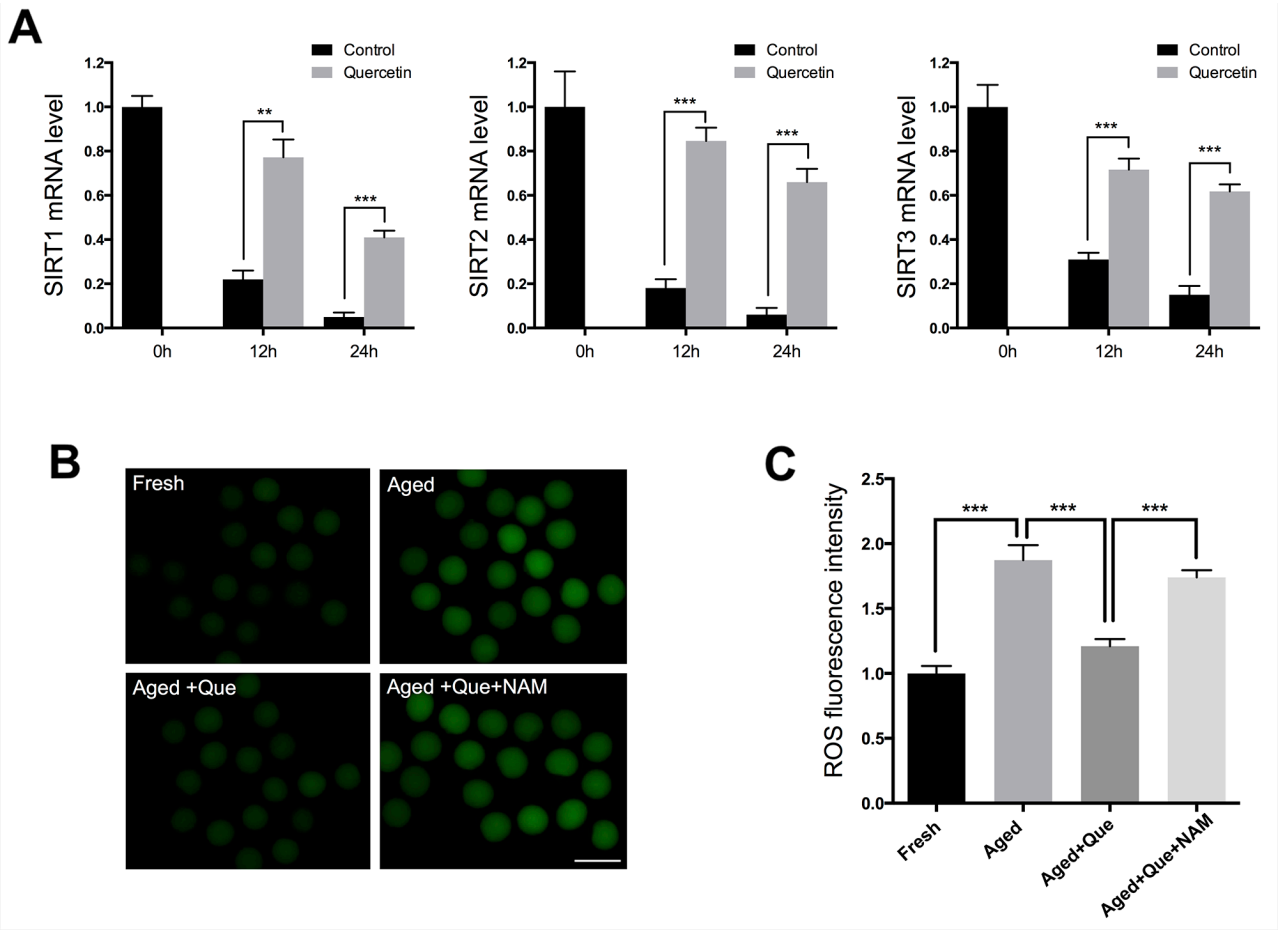
Effects of quercetin on SIRT1, 2, and 3 expression during postovulatory aging **(A)** Expression of SIRT1, 2, and 3 mRNA in control or quercetin-treated MII oocytes. MII oocytes were collected after culturing for 0, 12, and 24 h in the presence or absence of 10 μM quercetin. **(B)** Representative images of ROS fluorescence in MII oocytes. Oocytes were cultured for 12 h in M16 medium for aging. Scale bar: 100 μm. **(C)** Quantitative analysis of ROS fluorescence intensity. ***p < 0.001. Que: quercetin.

A decrease in the expression levels of SIRT1, 2, and 3 has recently been associated with a deterioration in oocyte quality during postovulatory aging [11]. This finding led us to hypothesize that quercetin delays oocyte aging by preventing a decrease in SIRT expression. To explore the potential correlation between quercetin and SIRT during oocyte aging, we examined SIRT expression following quercetin treatment. Real-time RT-PCR analysis revealed that the mRNA expression levels of SIRT1, 2, and 3 were all markedly reduced with oocyte aging (Figure 3A). However, treatment with 10 μM quercetin prevented the decrease in SIRT1, 2, and 3 during the oocyte aging process (Figure 3A). Consistent with this finding, inhibition of SIRT activity with nicotinamide (NAM) reversed the preventive effect of quercetin on ROS generation (Figure 3B and 3C). Therefore, our results suggest that quercetin prevents the decrease in SIRT expression during postovulatory aging.

**Figure 4:**
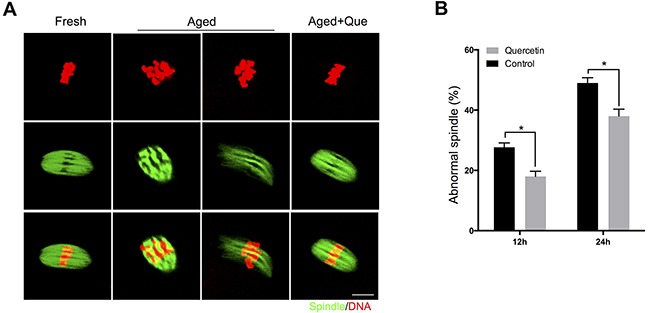
Effects of quercetin on spindle morphology during postovulatory aging **(A)** Representative images of spindle morphology in fresh, aged, and quercetin-treated MII oocytes. Scale bar: 10 μm. Que: quercetin. **(B)** The proportion of abnormal spindles in control or 10 μM quercetin-treated oocytes after 12 h or 24 h of aging, respectively. Data are expressed as mean ± SEM of at least three independent experiments. *p < 0.05.

Because inhibition of SIRT1, 2, and 3 has been shown to increase the frequency of spindle defects [11], we analyzed whether the quercetin-mediated delay in SIRT1, 2, and 3 downregulation could rescue these phenotypes of aging oocytes. MII oocytes were exposed to quercetin for 12 h or 24 h and then immunolabeled for analysis of spindle organization and mitochondrial distribution. As shown in Figure 4A, fresh oocytes displayed typical barrel-shaped spindles. However, in aged oocytes, the spindles became elongated, the microtubules were gradually lost from the spindle, and aberrant chromosome alignment was increased. Analysis of MII oocytes after 12 h or 24 h of aging showed that the proportion of abnormal spindles was significantly reduced in the quercetin-treated group compared to the control group (Figure 4B).

**Figure 5:**
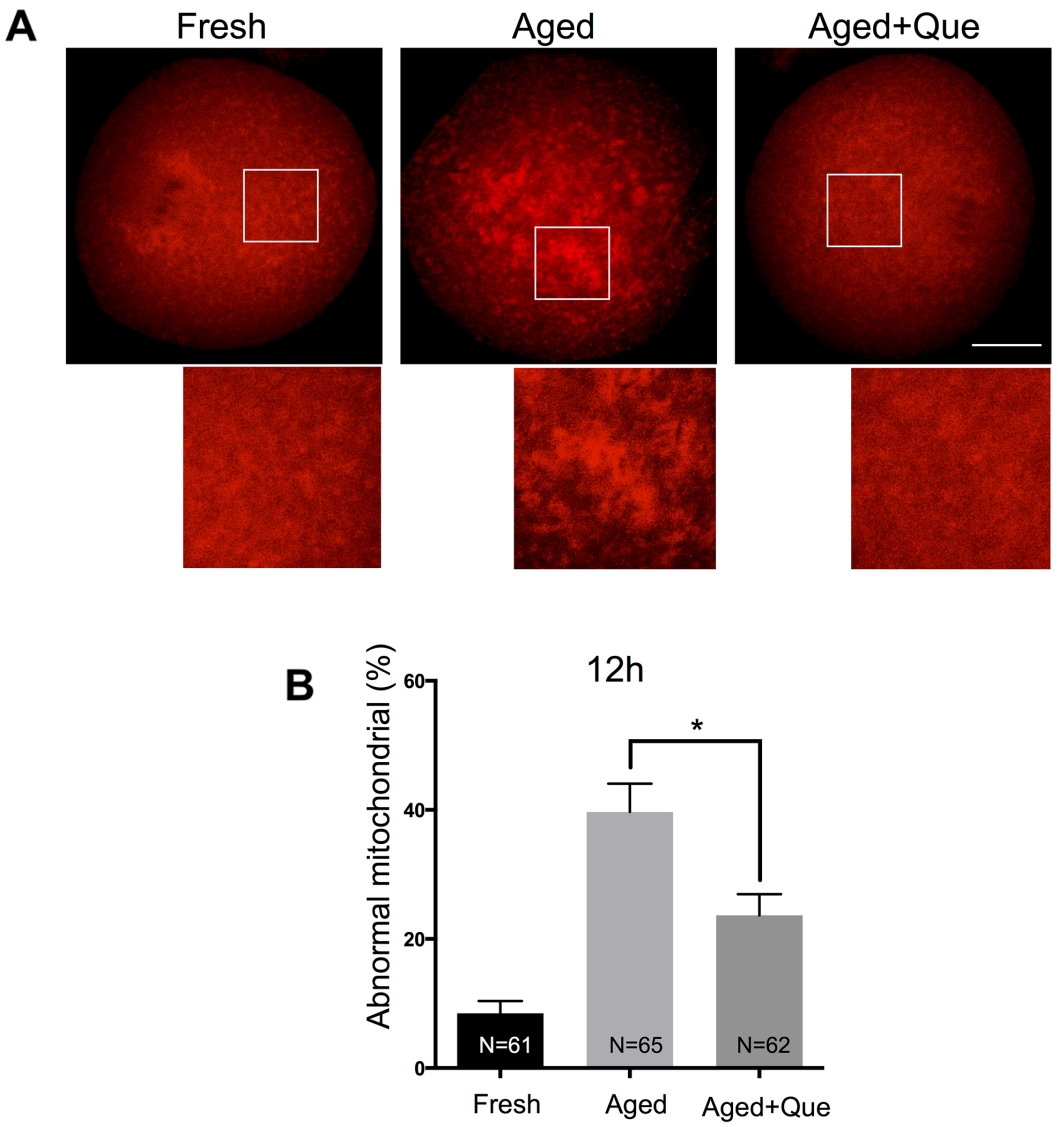
Effects of quercetin on mitochondrial distribution during postovulatory aging **(A)** Representative images of mitochondrial distribution in fresh, aged, and quercetin-treated MII oocytes. Scale bar: 20 μm. Que: quercetin. **(B)** The proportion of abnormal mitochondrial morphology in control oocytes or quercetin-treated oocytes after 12 h and 24 h of aging, respectively. Data are expressed as mean ± SEM of at least three independent experiments. *p < 0.05.

Because mitochondria are also considered to be an important subcellular target of SIRT1 under oxidative stress, we next examined whether mitochondrial distribution was affected by quercetin treatment during postovulatory aging of oocytes. Whereas evenly distributed throughout the cytoplasm in fresh MII oocytes, mitochondria were aggregated in large clusters in aged MII oocytes. However, these abnormal distribution patterns were significantly reduced in oocytes treated with quercetin (Figure 5A and 5B). These results suggest that the quercetin-induced delay of SIRT1, 2, and 3 downregulation prevents aging-associated changes in oocytes.

### Quercetin reduces loss of H3K9me3 during oocyte aging

**Figure 6:**
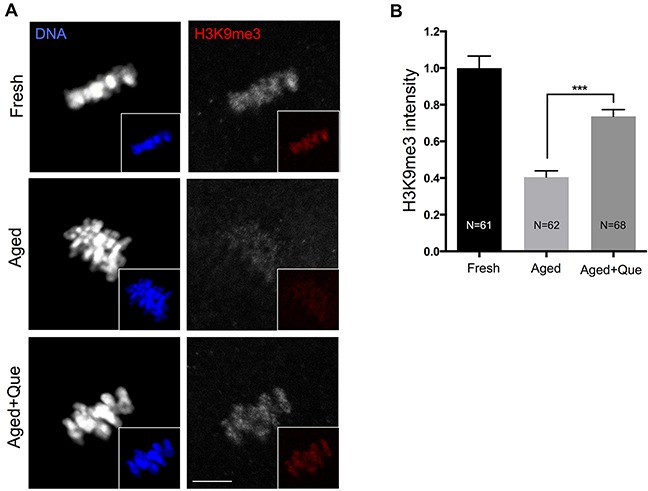
Effects of quercetin on histone H3K9 trimethylation during postovulatory aging **(A)** Representative images of histone H3K9 trimethylation in fresh, aged, and quercetin-treated MII oocytes. Scale bar: 10 μm. **(B)** Quantitative analysis of H3K9me3 fluorescence intensity. Oocytes were cultured for 12 h with or without 10 μM of quercetin for aging. ***p < 0.001. Data are expressed as mean ± SEM of at least three independent experiments. Que: quercetin.

Since SIRT1 induces trimethylation of histone H3 at lysine 9 (H3K9me3) by increasing the activity of histone methyltransferase SUV39H1 [19], it was of interest to examine whether quercetin preserves the methylation patterns of histones during postovulatory oocyte aging. Analysis of H3K9 methylation in fresh and postovulatory aged oocytes showed that H3K9me3 was decreased after 12 h of oocyte aging, which was consistent with a previous study reporting that methylation patterns were disrupted with oocyte aging [20]. However, H3K9me3 patterns were restored when oocytes were treated with quercetin (Figure 6A, 6B). These results suggest that the quercetin-induced delay of SIRT1, 2, and 3 downregulation prevents histone modification during postovulatory oocyte aging.

### Quercetin prevents a decrease in MPF activity during postovulatory aging

**Figure 7:**
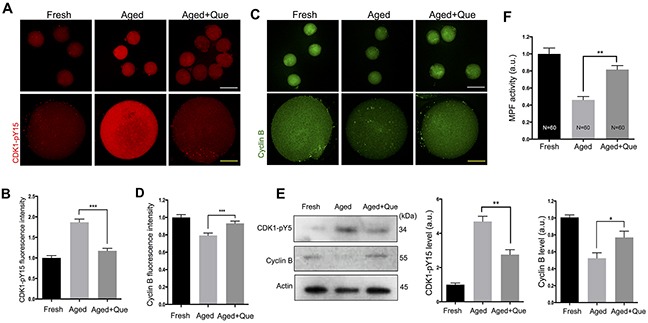
Effects of quercetin on MPF activity during postovulatory aging Oocytes were cultured for 12 h with or without 10 μM of quercetin. **(A)** Representative images showing changes in CDK1-pY15 levels in fresh, aged, and quercetin-treated MII oocytes. Scale bars: 20 μm (Yellow), 100 μm (White). **(B)** Quantitative analysis of CDK1-pY15 fluorescence intensity. **(C)** Representative images showing changes in cyclin B levels. Scale bars: 20 μm (Yellow), 100 μm (White). **(D)** Quantitative analysis of cyclin B fluorescence intensity. **(E)** The expression levels of CDK1-pY15 and cyclin B were determined by immunoblot analyses. The normalized levels of CDK1-pY15 and cyclin B are shown. **(F)** MPF activity was measured in MII oocytes after 12 h of *in vitro* culture. *p < 0.05, **p < 0.01, ***p < 0.001. Data are expressed as mean ± SEM of at least three independent experiments. Que: quercetin.

MPF is important for maintaining oocyte arrest at the MII stage, and a gradual decline in its activity has been detected in postovulatory aging [21]. Moreover, decreased MPF activity has been associated with increased levels of parthenogenetic activation and fragmentation [21]. Because quercetin treatment reduced aging-associated morphological changes such as parthenogenetic activation and fragmentation (Figure 1), we hypothesized that quercetin treatment may also prevent the loss of MPF activity as another mechanism contributing to delayed oocyte aging. To test this hypothesis, we measured MPF activity by examining the phosphorylation status of CDK1 at Tyr-15 (CDK1-pY15) and the expression levels of cyclin B. Immunostaining analysis revealed that after 12 h *in vitro* culture of oocytes, the level of CDK1-pY15 was increased, whereas the level of cyclin B expression was decreased. However, quercetin treatment significantly reduced the upregulation of CDK1-pY15 and the downregulation of cyclin B during *in vitro* oocyte culture (Figure 7A–7D). The quercetin-mediated rescue of CDK1-pY15 and cyclin B levels was further confirmed by immunoblot analysis (Figure 7E). In line with the increased CDK1-pY15 and decreased cyclin B levels, we found that MPF activity was decreased during oocyte aging. However, this decrease was significantly reduced after quercetin treatment (Figure 7F). Therefore, our results suggest that quercetin can delay oocyte aging by preserving MPF activity.

### Quercetin delays the onset of apoptosis during postovulatory aging of oocytes

**Figure 8:**
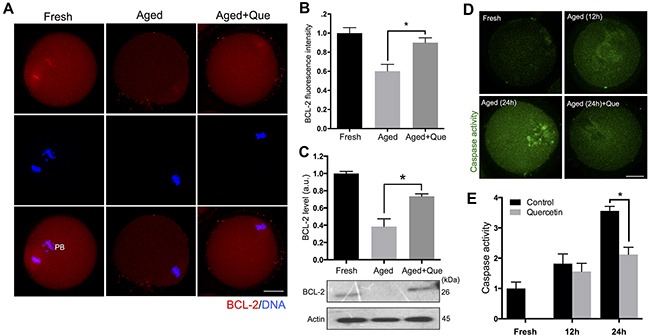
Effects of quercetin on oocyte apoptosis during postovulatory aging **(A)** Representative images showing changes in BCL-2 levels in fresh, aged, and quercetin-treated MII oocytes. Scale bar: 20 μm. **(B)** Quantitative analysis of BCL-2 fluorescence intensity. Oocytes were cultured for 12 h with or without 10 μM of quercetin for aging. **(C)** The expression levels of BCL-2 were determined by immunoblot analyses. The normalized levels of BCL-2 are shown. Oocytes were cultured for 12 h with or without 10 μM of quercetin for aging. **(D)** Representative images showing changes of caspase activity in fresh, aged, and quercetin-treated MII oocytes. Scale bar: 20 μm. **(E)** Quantitative analysis of fluorescence intensity of caspase activation. Oocytes were cultured for 12 h and 24 h with or without 10 μM of quercetin. *p < 0.05. Data are expressed as mean ± SEM of at least three independent experiments. Que: quercetin.

Since the postovulatory aging process culminates in apoptosis by decreasing the expression of anti-apoptotic factor BCL-2 and activating caspase-3 [5, 22, 23], it is possible that quercetin reduces the level of apoptosis. Immunostaining analysis revealed that BCL-2 expression decreased during postovulatory oocyte aging. However, quercetin treatment significantly reduced this downregulation of BCL-2 expression during *in vitro* culture of oocytes (Figure 8A, 8B). Immunoblot analysis further confirmed the rescue of BCL-2 levels following quercetin treatment (Figure 8C). Moreover, we observed a significant rise in the level of caspase activation during postovulatory aging of oocytes (Figure 8D). Similar to the BCL-2 results, the level of caspase activation was significantly reduced after quercetin treatment at 24 h of culture (Figure 8E). These results suggest that quercetin delays the onset of apoptosis during postovulatory aging of oocytes.

### Quercetin improves the developmental potential of aged oocytes

**Figure 9:**
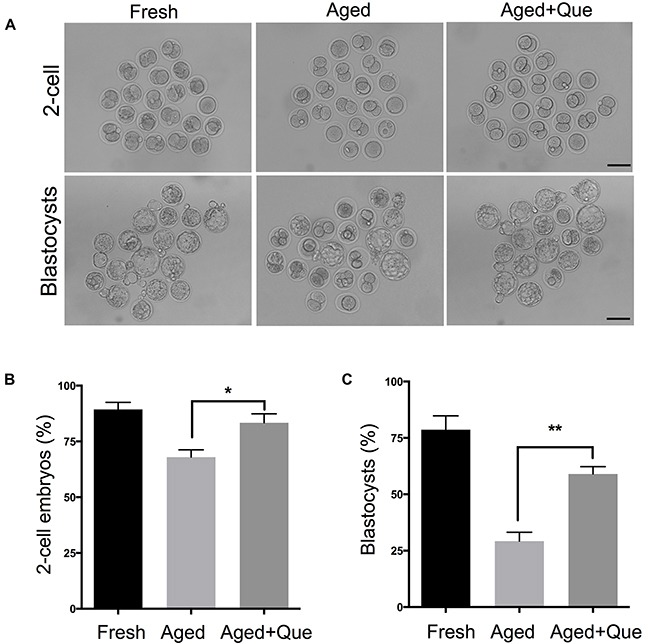
Quercetin improves preimplantation embryo development Oocytes treated with 10 μM of quercetin or DMSO were inseminated by ICSI after 12 hours of *in vitro* culture. As a control, fresh oocytes were inseminated by ICSI. **(A)** Representative images of 2-cell embryos and blastocysts are shown. Scale bar: 100 μm. **(B, C)** The rate of 2-cell and blastocyst development was shown. *p < 0.05, **p < 0.001. Que: quercetin.

To extend our findings that quercetin ameliorates aging-associated decline in oocyte quality, we performed intracytoplasmic sperm injection on aged oocytes. Consistent with quercetin-mediated improved oocyte quality, the rate of 2-cell and blastocyst development was significantly increased after quercetin treatment (Figure 9A–9C). Therefore, our data show that quercetin effectively improves embryo development.

## DISCUSSION

Oxidative stress acts as a ‘trigger’ for a cascade of other factors that orchestrate postovulatory aging, as well as directly affecting multiple aspects of oocyte biochemistry and functionality [5, 7, 24]. Increased oxidative stress in postovulatory aged oocytes is due to a progressive increase in ROS production and the concomitant depletion of antioxidant protection, as well as environmental factors [7]. Previous studies have shown that ROS generation during postovulatory oocyte aging is a key factor that influences oocyte quality [2, 7]. Here, we observed that ROS accumulation was significantly reduced when oocytes were treated with quercetin. Consistent with this finding, early mouse embryos treated with quercetin exhibited decreased H_2_O_2_-induced intracellular ROS levels [18], suggesting that quercetin exerts antioxidant effects at the both the postovulatory aging process and subsequent early embryo development.

Given that the SIRT1, 2, and 3 pathways protected MII oocytes against aging by reducing ROS generation [11], we speculated that quercetin may preserve oocyte quality by preventing the decrease in SIRT expression. Consistent with this, the decrease of SIRT1, 2, and 3 expression was significantly reduced in oocytes after quercetin treatment. These results demonstrate that quercetin enhances SIRT1, 2, and 3 expression in postovulatory aged oocytes, which is in concordance with previous studies reporting that quercetin increased SIRT1 expression in somatic cells [25, 26]. Moreover, we used NAM (a SIRT1, 2, and 3 inhibitor) to investigate the correlation between SIRT activity and quercetin. As expected, NAM efficiently inhibited the beneficial effect of quercetin on ROS scavenging, suggesting that the effect of quercetin on ROS clearance is SIRT-dependent. Recently, SIRT1, 2, and 3 were shown to be important for spindle organization and mitochondrial distribution in mouse oocytes [11, 12, 25, 27]. We also found that quercetin treatment reduced the frequency of spindle defects and altered the mitochondrial distribution, further supporting our conclusion that quercetin protects oocytes from postovulatory oocyte aging in a SIRT-dependent manner.

The level of H3K9me3 has been reported to be decreased in oocytes aged *in vitro* [9, 28]. Given that SIRT1 is a NAD^+^-dependent histone deacetylase that increases the levels of H3K9me3 through a SUV39H1-dependent mechanism [19, 20], our results suggest that quercetin prevents the decrease of H3K9me3 levels by maintaining SIRT expression during postovulatory aging of oocytes. However, the detailed mechanisms underlying the interplay between quercetin and SIRT in postovulatory oocytes deserve further investigation.

Declines in MPF activity during postovulatory aging often cause spontaneous exit from MII arrest, leading to parthenogenetic activation and fragmentation. Because quercetin reduced aging-associated morphological changes, including parthenogenetic activation and fragmentation, it is possible that quercetin could maintain MPF activity during postovulatory oocyte aging. Indeed, we found that MPF activity was significantly enhanced when oocytes were treated with quercetin during postovulatory aging. Consistent with our results, quercetin treatment has been reported to increase cyclin B1 level and CDK1 kinase activity in cancer cells [29]. Therefore, quercetin appear to possess properties that maintain MPF activity, thereby preventing parthenogenesis and fragmentation in ovulated oocytes.

Accumulation of ROS and decreased MPF activity have been shown to activate apoptotic pathways [5, 7, 8]. Given that the end point of oocyte aging is cell death via an apoptotic pathway [8], it is reasonable to speculate that quercetin suppresses the activation of apoptotic pathway. We found that quercetin delayed the onset of apoptosis, as demonstrated by the increased accumulation of BCL-2 and decreased caspase activity after quercetin treatment. Therefore, it is likely that quercetin delays the onset of apoptosis by decreasing SIRT-dependent ROS generation and maintaining MPF activity during postovulatory oocyte aging.

In summary, our data show that quercetin treatment prevents multiple changes associated with oocyte aging and apoptosis, thereby improving subsequent early embryo development.

## MATERIALS AND METHODS

### Antibodies and chemicals

All chemicals used in this study were purchased from Sigma-Aldrich unless otherwise stated. Mouse monoclonal anti-α-tubulin FITC-conjugated antibodies and Hoechst 33342 were from Sigma (St. Louis, MO, USA). Goat polyclonal anti-CDK1 (phospho Y15) antibodies and goat polyclonal anti-cyclin B antibodies were from Santa Cruz Biotechnology (Dallas, TA, USA). Rabbit polyclonal anti-H3K9me3 antibodies were from Abcam (Cambridge, MA, USA). Rabbit polyclonal anti-BCL-2 antibodies were from Cell Signaling Technology (Danvers, MA, USA).

### Oocyte collection and culture

Six-week-old ICR female mice were used in the study. All animal care and handling procedures were conducted in accordance with the guidelines regarding the care and use of animals issued by the ethics committee of the Department of Animal Science, Chungbuk National University. In addition, all experimental protocols were approved by the Animal Research Committee, Chungbuk National University (approval no. CBNUR-889-15-02).

To obtain fresh MII oocytes, 5 IU of human chorionic gonadotrophin (hCG, Sigma) were administered 48 h after PMSG injection. Oocytes were collected in M2 medium at 13-14 h after hCG injection, and cumulus cells were removed using hyaluronidase. After washing, cumulus-free oocytes were cultured in M16 medium in a 5% CO_2_ atmosphere at 37°C with or without quercetin.

### Oocyte aging and treatment

For *in vitro* aging, MII oocytes were cultured in 20 μl droplets of M16 medium under mineral oil for 12 or 24 h with or without quercetin treatment. Quercetin and nicotinamide were dissolved in DMSO, and quercetin was then diluted to a final concentration of 1, 5, or 10 μM with M16 medium. Nicotinamide was diluted with M16 medium to a final concentration of 10 μM. For both treatments, the final concentration of the solvent did not exceed 0.05% of the culture medium.

### Detection of intracellular ROS levels

To determine the levels of intracellular ROS production, oocytes were incubated for 30 min at 37°C in M2 medium supplemented with 10 mM 2′,7′-dichlorodihydrofluorescein diacetate (H2DCFDA; Sigma-Aldrich). After washing, oocytes were observed under a Nikon Eclipse Ti inverted microscope with identical settings. Images were quantified using ImageJ software (National Institutes of Health, Bethesda, MD, USA).

### Immunofluorescence staining

Oocytes were fixed in 4% paraformaldehyde dissolved in PBS (pH 7.4) for 30 min and then permeabilized in 0.5% Triton X-100 for 20 min. After 1 h in blocking buffer (PBS containing 1% BSA), the oocytes were incubated overnight at 4°C with primary antibodies (1:50-1:100 dilution), followed by incubation with Alexa Fluor 488-conjugated or Alex Fluor 594-conjugated secondary antibodies (1:200, Sigma-Aldrich) for 1–2 h at room temperature. Hoechst 33342 (10 mg/mL in PBS) was used for DNA counterstaining. For mitochondrial staining, oocytes were incubated for 30 min at 37°C in M2 medium supplemented with 200 nM MitoTracker Red (Invitrogen, Carlsbad, CA, USA). Next, the oocytes were mounted on glass slides and examined under a laser scanning confocal microscope (Zeiss LSM 780 META, Oberkochen, Germany). At least 20 oocytes were examined in each group, unless stated otherwise.

For measurement of immunofluorescent intensity, the signals from both control and experimental oocytes were acquired by performing the same immunostaining procedure and setting up the same parameters of confocal microscope. Data were analyzed by Image J software.

### Immunoblotting analysis

In total, 200 mouse oocytes per sample were mixed with 2× SDS sample buffer and boiled for 5 min at 100°C for SDS-PAGE. Western blotting was performed as described previously [30, 31], using the following antibody dilutions: anti-BCL-2, 1:500; anti-CDK1-pY15, 1:500; anti-cyclin B, 1:500; anti-β-actin, 1:2000.

### Real-time RT-PCR analysis

Total RNA was extracted from 100 oocytes using a Dynabead mRNA DIRECT^TM^ Kit (Ambion, Austin, TX, USA). First-strand cDNA was generated using a cDNA synthesis kit (LeGene, San Diego, CA, USA). GAPDH was selected as a reference gene. Primer sequences are shown in Supplementary Table 1. The KAPA SYBR® FAST qPCR Kits (Kapa Biosystems, Wilmington, MA, USA) were used in combination with a CFX Connect™ Real-Time PCR Detection System (Bio-Rad Laboratories, Hercules, CA, USA). Relative gene expression was calculated by the 2^ΔΔCt^ method.

### Caspase assay

A FAM-FLICA Poly Caspase Assay Kit (ImmunoChemistry Technologies, LLC, Bloomington, MN, USA) was used to measure the level of caspase activation in oocytes according to the manufacturer's protocols. In brief, oocytes were incubated with the reaction mix in a humidified atmosphere of 5% CO2 at 39°C for 30 min. After incubation, oocytes were washed three times in PBS-PVA.

### MPF kinase assay

Twenty oocytes per sample were lysed in 5 μL of lysis buffer (50 mM Tris-HCl [pH 7.5], 120 mM NaCl, 5 mM EGTA, 0.01% Brij-35 [Santa Cruz Biotechnology], 1 mM PMSF, 0.05 mg/mL leupeptin, 50 mM β-mercaptoethanol, 25 mM β-glycerophosphate, and 1 mM Na_3_VO_4_). The resultant lysates were stored at −80°C until use in the kinase assay. The kinase reaction was initiated by the addition of 45 μL of kinase buffer (25 mM 4-[2-hydroxyethyl]-1-piperazineethanesulfonic acid [HEPES] buffer [pH 7.5], 10 mM MgCl2, 10% [v/v] MV peptide solution [SLYSSPGGAYC; MBL International, Woburn, MA, USA], and 0.1 mM ATP). The reaction was allowed to proceed for 30 min at 30°C and was terminated by the addition of 200 μL of PBS containing 50 mM EGTA. Phosphorylated MV peptides were detected using an ELISA MESACUP Cdc2/Cdk1 Kinase Assay Kit (MBL International).

### Intracytoplasmic sperm injection (ICSI)

ICSI was performed as described previously [32]. Briefly, sperm were selected randomly and the sperm heads were separated from the tails using a high Piezo pulse before they were injected singly into each oocyte. After 10 min of recovery at room temperature, the oocytes were cultured in KSOM medium in a 5% CO2 atmosphere at 37°C. In these experiments, aged oocytes were used only if they had normal surface features and were not activated spontaneously. After 6-8 h of culture, pronuclear formation was observed. The developmental competence of fresh and aged oocytes was evaluated based on the percentage of sperm injected and survived oocytes that reached to 2-cell and blastocyst stage after 22-24 and 96-98 h, respectively.

### Statistical analysis

Statistical analysis was performed using GraphPad Prism (GraphPad Software, La Jolla, CA, USA). All experiments were repeated at least three times unless specified otherwise, and each experimental group included at least 20 oocytes. The significance of differences between groups was analyzed by Student's *t*-test. Data are expressed as mean ± SEM; differences with p values < 0.05 were considered statistically significant.

## SUPPLEMENTARY TABLES





## References

[1] Miao YL, Kikuchi K, Sun QY, Schatten H (2009). Oocyte aging: cellular and molecular changes, developmental potential and reversal possibility. Hum Reprod Update.

[2] Takahashi T, Takahashi E, Igarashi H, Tezuka N, Kurachi H (2003). Impact of oxidative stress in aged mouse oocytes on calcium oscillations at fertilization. Mol Reprod Dev.

[3] Wakayama S, Thuan NV, Kishigami S, Ohta H, Mizutani E, Hikichi T, Miyake M, Wakayama T (2004). Production of offspring from one-day-old oocytes stored at room temperature. J Reprod Dev.

[4] Xu Z, Abbott A, Kopf GS, Schultz RM, Ducibella T (1997). Spontaneous activation of ovulated mouse eggs: time-dependent effects on M-phase exit, cortical granule exocytosis, maternal messenger ribonucleic acid recruitment, and inositol 1,4,5-trisphosphate sensitivity. Biol Reprod.

[5] Lord T, Nixon B, Jones KT, Aitken RJ (2013). Melatonin prevents postovulatory oocyte aging in the mouse and extends the window for optimal fertilization in vitro. Biol Reprod.

[6] Ducibella T, Duffy P, Reindollar R, Su B (1990). Changes in the distribution of mouse oocyte cortical granules and ability to undergo the cortical reaction during gonadotropin-stimulated meiotic maturation and aging in vivo. Biol Reprod.

[7] Lord T, Aitken RJ (2013). Oxidative stress and ageing of the post-ovulatory oocyte. Reproduction.

[8] Tatone C, Carbone MC, Gallo R, Delle Monache S, Di Cola M, Alesse E, Amicarelli F (2006). Age-associated changes in mouse oocytes during postovulatory in vitro culture: possible role for meiotic kinases and survival factor BCL2. Biology of reproduction.

[9] Ge ZJ, Schatten H, Zhang CL, Sun QY (2015). Oocyte ageing and epigenetics. Reproduction.

[10] Prasad S, Koch B, Chaube SK (2016). RO-3306 prevents postovulatory aging-mediated spontaneous exit from M-II arrest in rat eggs cultured in vitro. Biomed Pharmacother.

[11] Zhang T, Zhou Y, Li L, Wang HH, Ma XS, Qian WP, Shen W, Schatten H, Sun QY (2016). SIRT1, 2, 3 protect mouse oocytes from postovulatory aging. Aging.

[12] Hung CH, Chan SH, Chu PM, Tsai KL (2015). Quercetin is a potent anti-atherosclerotic compound by activation of SIRT1 signaling under oxLDL stimulation. Mol Nutr Food Res.

[13] Casella ML, Parody JP, Ceballos MP, Quiroga AD, Ronco MT, Frances DE, Monti JA, Pisani GB, Carnovale CE, Carrillo MC, de Lujan Alvarez M (2014). Quercetin prevents liver carcinogenesis by inducing cell cycle arrest, decreasing cell proliferation and enhancing apoptosis. Mol Nutr Food Res.

[14] Liu K, Mei F, Wang Y, Xiao N, Yang L, Wang Y, Li J, Huang F, Kou J, Liu B, Qi LW (2016). Quercetin oppositely regulates insulin-mediated glucose disposal in skeletal muscle under normal and inflammatory conditions: The dual roles of AMPK activation. Mol Nutr Food Res.

[15] Li Y, Yao J, Han C, Yang J, Chaudhry MT, Wang S, Liu H, Yin Y (2016). Quercetin Inflammation and Immunity. Nutrients.

[16] Kang JT, Moon JH, Choi JY, Park SJ, Kim SJ, Saadeldin IM, Lee BC (2016). Effect of Antioxidant Flavonoids (Quercetin and Taxifolin) on In vitro Maturation of Porcine Oocytes. Asian-Australas J Anim Sci.

[17] Kang JT, Kwon DK, Park SJ, Kim SJ, Moon JH, Koo OJ, Jang G, Lee BC (2013). Quercetin improves the in vitro development of porcine oocytes by decreasing reactive oxygen species levels. J Vet Sci.

[18] Yu S, Long H, Lyu QF, Zhang QH, Yan ZG, Liang HX, Chai WR, Yan Z, Kuang YP, Qi C (2014). Protective effect of quercetin on the development of preimplantation mouse embryos against hydrogen peroxide-induced oxidative injury. PLoS One.

[19] Vaquero A, Scher M, Erdjument-Bromage H, Tempst P, Serrano L, Reinberg D (2007). SIRT1 regulates the histone methyl-transferase SUV39H1 during heterochromatin formation. Nature.

[20] Manosalva I, Gonzalez A (2010). Aging changes the chromatin configuration and histone methylation of mouse oocytes at germinal vesicle stage. Theriogenology.

[21] Kikuchi K, Naito K, Noguchi J, Shimada A, Kaneko H, Yamashita M, Aoki F, Tojo H, Toyoda Y (2000). Maturation/M-phase promoting factor: a regulator of aging in porcine oocytes. Biol Reprod.

[22] Fujino Y, Ozaki K, Yamamasu S, Ito F, Matsuoka I, Hayashi E, Nakamura H, Ogita S, Sato E, Inoue M (1996). DNA fragmentation of oocytes in aged mice. Hum Reprod.

[23] Gordo AC, Rodrigues P, Kurokawa M, Jellerette T, Exley GE, Warner C, Fissore R (2002). Intracellular calcium oscillations signal apoptosis rather than activation in in vitro aged mouse eggs. Biol Reprod.

[24] Lord T, Martin JH, Aitken RJ (2015). Accumulation of electrophilic aldehydes during postovulatory aging of mouse oocytes causes reduced fertility, oxidative stress, and apoptosis. Biol Reprod.

[25] Zhang L, Hou X, Ma R, Moley K, Schedl T, Wang Q (2014). Sirt2 functions in spindle organization and chromosome alignment in mouse oocyte meiosis. FASEB J.

[26] Di Emidio G, Falone S, Vitti M, D'Alessandro AM, Vento M, Di Pietro C, Amicarelli F, Tatone C (2014). SIRT1 signalling protects mouse oocytes against oxidative stress and is deregulated during aging. Hum Reprod.

[27] Zhao LR, Du YJ, Chen L, Liu ZG, Pan YH, Liu JF, Liu B (2014). Quercetin protects against high glucose-induced damage in bone marrow-derived endothelial progenitor cells. International journal of molecular medicine.

[28] Trapphoff T, Heiligentag M, Dankert D, Demond H, Deutsch D, Frohlich T, Arnold GJ, Grummer R, Horsthemke B, Eichenlaub-Ritter U (2016). Postovulatory aging affects dynamics of mRNA, expression and localization of maternal effect proteins, spindle integrity and pericentromeric proteins in mouse oocytes. Hum Reprod.

[29] Kuo PC, Liu HF, Chao JI (2004). Survivin and p53 modulate quercetin-induced cell growth inhibition and apoptosis in human lung carcinoma cells. The Journal of biological chemistry.

[30] Wang H, Luo Y, Zhao MH, Lin Z, Kwon J, Cui XS, Kim NH (2016). DNA double-strand breaks disrupted the spindle assembly in porcine oocytes. Mol Reprod Dev.

[31] Wang H, Kim NH (2016). CDK2 Is Required for the DNA Damage Response During Porcine Early Embryonic Development. Biol Reprod.

[32] Kimura Y, Yanagimachi R (1995). Intracytoplasmic sperm injection in the mouse. Biol Reprod.

