# Supplementary material for: Quercetin delays postovulatory aging of mouse oocytes by regulating SIRT expression and MPF activity
Source: Oncotarget. 2017 Mar 15;8(24):38631–41. doi: 10.18632/oncotarget.16219 (PMC5503559; doi:10.18632/oncotarget.16219)
Supplement: Supplementary file 1 [file oncotarget-08-38631-s001.pdf]

Quercetin delays postovulatory aging of mouse oocytes by regulating SIRT expression and MPF activity

SUPPLEMENTARY TABLE

Supplementary Table 1: Primers used for real-time reverse transcription-PCR.

See Supplementary File 1
